# Complete Genome Sequence of *Bacillus megaterium* Siphophage Stills

**DOI:** 10.1128/genomeA.00855-15

**Published:** 2015-08-06

**Authors:** Simon S. Lee, Rohit R. Kongari, Adriana C. Hernandez, Gabriel F. Kuty Everett

**Affiliations:** Center for Phage Technology, Texas A&M University, College Station, Texas, USA

## Abstract

*Bacillus megaterium* is a soil-dwelling bacterium frequently used in research as a model organism and in industry in protein production applications. Bacteriophages may be used to enhance the use of this bacterium. Here, we describe the complete genome of *B. megaterium* siphophage Stills and its core features.

## GENOME ANNOUNCEMENT

The Gram-positive bacterium *Bacillus megaterium* is used as a model organism to study cell wall synthesis, membrane formation, and sporulation processes (1). It is also employed for large-scale protein production to synthesize penicillin and produce amylases for the baking industry (2). The study of phages infecting *B. megaterium*, such as siphophage Stills, described here, can aid in bioengineering to advance practical applications of this bacterium.

Bacteriophage Stills was isolated from a soil sample collected in College Station, TX. Phage DNA was sequenced in an Illumina MiSeq 250-bp paired-end run with a 550-bp insert library at the Genomic Sequencing and Analysis Facility at the University of Texas (Austin, TX, USA). Quality-controlled trimmed reads were assembled to a single contig at 38.1-fold coverage using Velvet version 1.2.10. The contig was confirmed to be complete by PCR using primers that face the upstream and downstream ends of the phage DNA. Products from the PCR amplification of the junctions of concatemeric molecules were sequenced by Sanger sequencing (Eton Bioscience, San Diego, CA). Genes were predicted using GeneMarkS (3) and corrected using software tools available on the Center for Phage Technology (CPT) Galaxy instance (https://cpt.tamu.edu/galaxy-public/). Morphology was determined using transmission electron microscopy performed at the Texas A&M University Microscopy and Imaging Center.

Stills contains an 80,798-bp double-stranded DNA (dsDNA) genome with a coding density of 90.2% and a GC content of 35.49%. It shares 74.7% and 76.4% nucleotide sequence identities with *B. megaterium* siphophages Slash (GenBank accession number NC_022774) and Staley (GenBank accession number NC_022767), respectively, as determined by Emboss Stretcher (4). A 491-bp terminal repeat was identified by homology with Slash and Staley. Stills has as a host range that includes asporogenic *B. megaterium* strain Km Sp and WSH320.

Genes encoding DNA replication/recombination proteins were identified including helicase, DNA primase, Holliday junction resolvase, and two nucleases. Stills also contains genes that encode DNA biosynthesis proteins such as dUTPase, thymidylate synthase, guanylate kinase, and ribonucleoside-diphosphate reductase (alpha and beta subunits). Phage morphogenesis proteins include a tail fiber, tailspike, tape measure, and tape measure chaperones. The tape measure protein contains LysM (peptidoglycan binding) and lysozyme domains suggesting a mechanism for initial cell surface recognition and DNA entry into the host cell (5, 6). Genes involved in lysis were identified as well, such as an l-alanyl-d-glutamate peptidase, a secreted *N*-acetylmuramyl-l-alanine amidase, and a class II holin (7). Stills also encodes two RNA polymerase sigma factors.

The presence of an integrase-like protein and a DNA-binding protein with a P1 Ant1-like DNA-binding domain suggests that Stills may be a temperate phage. Ant1 antagonizes the repression of the master regulator C1 of bacteriophage P1 (8). Stills also encodes a secreted LysM, YkwD-like CAP domain (IPR014258)-containing protein. The CAP superfamily contains cysteine-rich secretory proteins, antigen 5, and pathogenesis-related proteins, although the role of this protein in the phage infection cycle is unknown.

### Nucleotide sequence accession number.

The genome sequence of phage Stills was contributed to GenBank under the accession number KP696448.
